# Lack of motor prediction, rather than perceptual conflict, evokes an odd sensation upon stepping onto a stopped escalator

**DOI:** 10.3389/fnbeh.2014.00077

**Published:** 2014-03-20

**Authors:** Hiroaki Gomi, Takeshi Sakurada, Takao Fukui

**Affiliations:** NTT Communication Science Laboratories, Nippon Telegraph and TelephoneAtsugi, Japan

**Keywords:** implicit postural control, visual motion, optic flow, conscious awareness, action attribution

## Abstract

When stepping onto a stopped escalator, we often perceive an “odd sensation” that is never felt when stepping onto stairs. The sight of an escalator provides a strong contextual cue that, in expectation of the backward acceleration when stepping on, triggers an anticipatory forward postural adjustment driven by a habitual and implicit motor process. Here we contrast two theories about why this postural change leads to an odd sensation. The first theory links the odd sensation to a lack of sensorimotor prediction from all low-level implicit motor processes. The second theory links the odd sensation to the high-level conflict between the conscious awareness that the escalator is stopped and the implicit perception that evokes an endogenous motor program specific to a moving escalator. We show very similar postural changes can also arise from reflexive responses to visual stimuli, such as contracting/expanding optic flow fields, and that these reflexive responses produce similar odd sensations to the stopped escalator. We conclude that the high-level conflict is not necessary for such sensations. In contrast, the implicitly driven behavioral change itself essentially leads to the odd sensation in motor perception since the unintentional change may be less attributable to self-generated action because of a lack of motor predictions.

## Introduction

We usually think of well-learned daily movements, such as reaching and walking, as being fully controlled by our action intentions. The sensory outcomes of our intended movements would be perceived as results of self-generated actions. Computational models emphasize that this process depends on prediction by internal models acquired through motor learning (Miall and Wolpert, 1996; Blakemore et al., 1998; Wolpert et al., 1998). Here we focus on a typical exception to this rule, which arises from an implicit sensorimotor program.

When stepping onto a stationary escalator, many people frequently experience unusual body and leg movements and an associated odd sensation. These peculiar bodily sensations are sometimes described as “clumsy” or “weird”, but are obviously different from the sensations associated with the simple motor-error detection that occasionally occurs in daily life (Fukui et al., 2009). Even though we completely understand the environmental state of the “stopped” escalator, we cannot avoid behaving clumsily and feeling an odd sensation before adaptation. Fukui et al. (2009) reported that stepping onto a stopped escalator causes several behavioral changes (forward body sway, altered leg-landing movement, and decreased hip-forwarding speed), and found from a statistical path analysis that the forward body sway dominantly leads to the odd sensation. They also showed that the postural changes are driven by an implicit sensorimotor program which is functional for the moving escalator, rather than by a shift of the center of gravity by irregular step heights or the entrance slope of the escalator. However, a remaining possibility—that the postural imbalance is caused by depth misperception due to the binocular miss-correspondence of the continuous tread-board stripes, as pointed out previously (Cohn and Lasley, 1990; Munck-Fairwood, 1992)—has not been rigorously examined.

Similar unintended postural changes were also observed in short-term locomotor adaptation to a mobile sled (Reynolds and Bronstein, 2003, 2004, 2006; Bunday et al., 2006; Bronstein et al., 2009), suggesting a general strong association between motor control and visual context, as examined in several visuomotor adaptation aftereffects (Anstis, 1995; Pelah and Barlow, 1996). In these studies, unintentional behavioral changes caused by short-term adaptations of endogenous sensorimotor programs were well described, but subjective sensations were not systematically covered. Specifically, in our context, it is not yet clear whether the mental event (or brain processing) of the endogenous and implicit motor program, which predictively stabilizes the posture when a person steps onto a moving escalator, is indispensable for inducing the unique sensation mentioned above. One possible hypothesis is that the odd sensation is a result of high-level conflicts between the conscious awareness that the escalator is stopped and the implicit perception that drives the endogenous motor program for a moving escalator. According to this hypothesis, the endogenous motor program is necessary for inducing the odd sensation. If this is true, postural changes caused by something other than the endogenous escalator-specific motor program could not result in the odd sensation. Examining this specific prediction would be particularly important in exploring the interaction between implicit perception and conscious awareness.

## Materials and methods

### Participants

Fifty-six participants (mean age = 27.1 years, SD = 5.4 years, range of 18–39, 27 females, 29 males, all right handed) took part in this experiment. To avoid potential biases in sensation and behavior in the first experiment (described below), we did not ask the participants before the experiment whether they had had an experience of stepping onto a stopped escalator. In other words, there was no selection bias in participant recruitment. None of the participants reported having any motor or visual disorders. The participants had normal or corrected-to-normal visual acuity. Informed consent was obtained from all participants after the nature of the technique was explained, which was approved by the NTT Communication Science Laboratories Research Ethics Committee.

### Experimental setup

**Figure 1:**
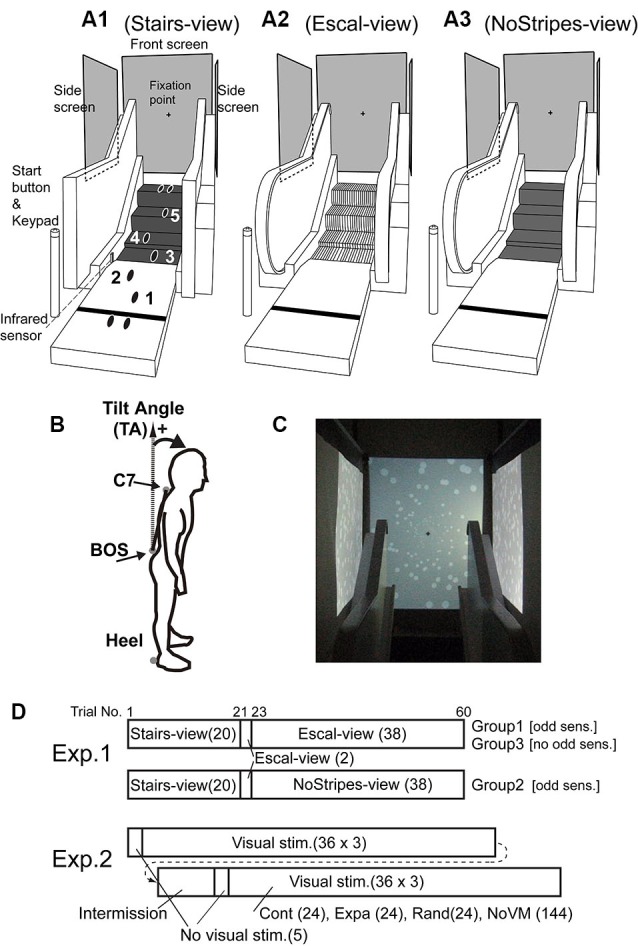
**Experimental setups using escalator mock-up**. **(A1)** Stairs-view condition, in which the handrail was covered with a black cloth with rectangular corners (to change the rounding shape of the handrail) and tread-boards were covered with black rubber sheets. **(A2)** Escal-view condition, in which the escalator aperture was fully viewed. **(A3)** NoStripes-view condition, in which tread-boards were veiled. Back-projected vertical screens were placed in the front and at bilateral sides. A fixation marker was shown around the center of the frontal screen during trials. **(B)** Marker locations for posture measurements [C7 of the spine, basis ossissacri (BOS), and right and left heels]. Tilt angle (TA) was calculated by the angle difference between the line C7 connecting BOS and the vertical axis. **(C)** White disk patterns for contraction/expansion/random motion stimuli on the frontal screen and for forward/backward/random motion stimuli on the bilateral screens. **(D)** Stimulus conditions throughout the trials in Experiments 1 and 2. Number of trials in each condition is indicated in parenthesis. In the first trial in the Escal-view condition, participants in Groups 1 and 2 reported the odd sensation, and participants in Group 3 did not. “Visual stimuli” in Experiment 2 consisted of static (NoVM), contraction (Cont), expansion (Expa), and random (Rand) stimulus conditions, randomly ordered.

In this experiment, we used an escalator mock-up that had four steps of a real escalator tread-board with an approach entrance (1.5 m) and bilateral hand rails (Figure 1A). The heights of the steps were 5 cm between the first and second steps, 19 cm between the second and third steps, and 20 cm between the third and fourth steps. The area of each tread-board was 60 (width) × 37 (depth) cm. Screens were placed on the frontal (width and height: 109 × 142 cm) and bilateral sides (135 × 100 cm) as shown in Figure 1A. All visual stimuli were programmed in Matlab (MathWorks, Natick, MA) with Cogent Graphics (University College London, London, UK) software on the Microsoft Windows (Seattle, WA) operating system, and were projected at 60 Hz (refresh rate) by computer projectors (WT610; NEC Viewtechnology). A trial-start button and a keypad for answering perception scores were placed in the vicinity of the start position (110 cm from the first tread-board of the escalator mock-up).

In the first experiment, we used three escalator appearance conditions: a fully veiled escalator condition (labeled as Stairs-view), in which tread-boards of the steps were covered by rubber sheets and hand-rails were occluded; an apparent escalator condition (labeled as Escal-view); and a veiled tread-board condition with the rubber sheets (labeled as NoStripes-view) (Figure 1A). A fixation marker (black cross 4 × 4 cm) was shown on the frontal screen with gray background, and side screens were filled by gray color. Reflexive 30-mm sphere markers were attached at four locations on the body: around C7 of the spine, basis ossissacri (BOS), and right and left heels (see Figure 1B). These markers were recorded with a three-dimensional motion capture system (ProReflex, Qualisys, Sweden) at a frequency of 250 Hz.

In the second experiment, we used four visual conditions. NoVM: static images of random white disks (luminance contrast of 0.6, diameters of 0.4–8 cm on the front and 4 cm on the side screens. See Figure 1C). Cont: moving white disk images of contraction (1.2 s for 90 cm from corner to center of the screen with exponential speed decrease) on the front screen and forward motion (1.0 cm/s) on the side screens. Expa: moving white disk images of expansion (exponential speed increase) on the front screen and backward motion (−1.0 cm/s) on the side screens. Rand: random replacements (noise like) of white disks at a rate of 30 Hz. The last three dynamic stimuli were triggered by a foot passing over an infrared sensor (Photoelectric Sensor PW-51, Keyence, Japan) just before the third step (see Figure 1A1, Stairs-view). The same four markers attached at the same body locations as in the first experiment were recorded.

### Experimental procedure

The first experiment was designed to examine the postural change and odd sensation when the tread-board stripes of the escalator were occluded. To fairly examine the effect, we first checked the odd sensation and postural tilt in the Stairs-view condition, and then characterized those in the Escal-view and NoStripes-view conditions. All participants (*n* = 56) were asked to stand straight at the start position (110 cm from the first step of the escalator mock-up) and initiate each trial by hitting the start button. Three beeps (interval of 0.52 s) and seven beeps (interval of 0.52 s) were successively given with insertion of 1.04 s pause. At the fourth beep, they started walking toward and stepping onto the fully veiled escalator mock-up (Stairs-view) while watching the fixation marker at the center of the front screen. Before the experiment, they were also asked to start gaits with the right leg and to place each foot as shown in Figure 1A1. After each trial in the Stairs-view condition (20 trials), participants were asked whether they had felt an odd sensation (yes/no) when climbing the steps.

After the Stairs-view condition, the escalator tread-board and escalator hand-belt were uncovered. All of the participants, upon being verbally asked by the experimenter whether they had been aware that the stairs was an escalator mock-up, indicated they had not.

Next, they were asked to step onto the apparent escalator mock-up (Escal-view) and to report whether they felt an odd sensation of body posture. When the participants felt some kind of odd sensation in the first trial in the Escal-view condition, we asked them to score the extent of the odd sensation after each trial on a five-point scale relative to the odd sensation in the initial trial in the Escal-view condition (5, same extent; 1, almost none). Even if the participants had not felt any kind of odd sensation in the first trial in the Escal-view condition, we asked them to step onto the escalator mock-up in order to measure their motor behavior. 48 participants felt an odd sensation in the first and second trials in the Escal-view condition, and the remaining eight participants did not. 29 out of the 48 participants (Group 1) continued the task in this condition (40 trials in total, including the two initial check trials). Eight participants who did not feel the odd sensation were designated as Group 3.

For 19 (Group 2) out of the 48 participants who reported an odd sensation for the first trial in the Escal-view condition, the tread-boards were veiled again (i.e., NoStripes-view condition) after two trials in the Escal-view condition. Note that, these two trials in the Escal-view condition before the NoStripes-view condition were carried out as a prescreening so that we could exclude participants who did not feel the odd sensation (Group-3 participants) in the stopped-escalator condition. This procedure was necessary to clearly examine the effect of the NoStripes-view condition for participants who felt the odd sensation in the Escal-view condition. Then the NoStripes-view condition (38 trials) was imposed to test the hypothesis that the cause of the postural change accompanied by the odd sensation on the stopped escalator is depth misperception due to the periodical surface grooves of the tread-boards. If this hypothesis is true, we can expect significant reductions of the postural change and the odd sensation in the NoStripes-view condition, compared to the Escal-view condition. Stimulus conditions throughout the trials in all participant groups are depicted in Figure 1D.

Second experiment was designed to examine whether the reflexively triggered postural change evokes an odd sensation similar to that induced by the endogenous implicit motor program. The 19 participants (5 from Group 1 and 14 from Group 2) who had reported the odd sensation in the first experiment took part in this experiment. The participants took more than a 20 min intermission after the first experiment. The tread-boards were uncovered throughout this experiment. They were asked to step onto the escalator mock-up and report the odd sensation score (36 trials/block, 6 blocks) on the five-point scale, as in the Escal-view and NoStripes-view conditions of the first experiment. They were also requested to report the subjective similarity [score of 1 (not similar) to 5 (strongly similar)] with the odd sensation experienced in the first experiment. The static visual patterns of white disks were shown on the screen (see Section Experimental Setup) at the start of each trial, and then these disks started to move according to the stimulus conditions (144 trials in NoVM, 24 trials in Cont, 24 trials in Expa, and 24 trials in Rand in 6 blocks, and stimuli were randomly ordered) when the infrared sensor detected the foot motion for the third step (see Section Experimental Setup). Note that, before the stimulus trials, the participants were asked to step onto the escalator mock-up five times without any white disk images to help them to recall the adapted behavior to the escalator mock-up. An intermission (15–20 min) was given after the first three blocks. The trial sequence of Experiment 2 is also shown in Figure 1D.

### Data analysis

For motion data analysis, each marker data point was filtered offline using a fourth-order Butterworth filter (double sided) with a cutoff frequency of 5 Hz and then differentiated to obtain velocity. As indices of postural sway, we used tilt angle (TA) defined as the angle made by the line from C7 connecting the BOS and gravitational line (Figure 1B). Heel contacts (HCs) on the floor or steps were detected by a heuristic algorithm using heel velocity and positions and confirmed visually in the heel temporal patterns. All behavioral data were temporally aligned at the HC of the third step. To focus on the upper body’s TA change as the steps were climbed, which could be affected by the visual stimuli, we calculated the difference between TAs at the third and fifth HCs (dTA), and then defined “dTA-diff” by subtracting the dTA value of the baseline condition (Stairs-view condition in Experiment 1 and NoVM condition in Experiment 2) from the dTA value of the target conditions.

The odd sensation scores reported on the five-point scale in the Escal-view and NoStripes-view conditions of the first experiment and all stimulus conditions in the second experiment were treated as the interval scale (Westermann, 1985). To examine condition-dependent differences and temporal changes in the odd sensation score and postural change (dTA-diff) in Experiment 1, the values of initial trials (23rd–27th) and late trials (56–60th) were respectively averaged for each participant, and then the mean values of odd sensation scores and postural change (dTA-diff) were respectively analyzed by a two-way repeated measures ANOVA with trial-phase (initial/late) and group (view conditions) as factors.

To examine the effect of each dynamic visual stimulus on the postural change (dTA-diff), the odd sensation score, and the odd sensation similarity score in Experiment 2, those values averaged over the first 10 trials of each stimulus condition were calculated, and then those variations were analyzed by a one-way repeated measures ANOVA with the stimulus condition (NoVM, Cont, Expa, and Rand) as the factor. Tukey’s HSD procedure was used for *post-hoc* comparison of means.

## Results

### Effects of visual appearance of escalator on behavioral change and odd sensation

The majority of the participants (48/56, 86%) reported a clear odd sensation when they first stepped onto the escalator mock-up (Escal-view condition), while they did not report such a sensation throughout all the trials without the view of the escalator hand-rail and tread-boards (Stairs-view condition). Note that remaining 8 participants in Group 3 reported lowest value of the odd sensation score throughout the sessions even in the Escal-view condition.

**Figure 2:**
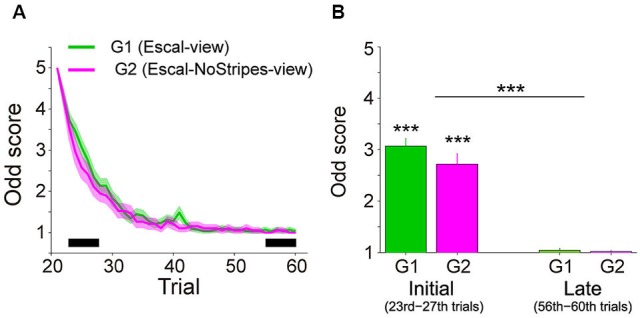
**Subjective scores of odd sensation over the trials in the Escal-view and the NoStripes-view conditions (A2 and A3 in Figure 1). (A)** Trial histories of odd sensation scores of G1 (Group 1: Escal-view, colored by green) and G2 (Group 2: Escal-NoStripes-view, colored by pink). Solid line: mean, Colored area: SE. **(B)** Comparison between mean scores during initial phase (23rd–27th) and late phase (56–60th), indicated by the thick horizontal bar in panel **A**, in both groups. Asterisks above the bar denote statistical significance of the mean from one by a *t*-test, and asterisks above the horizontal line denote statistical significance of the trial-phase (initial/late) factor, obtained by ANOVA. The error bar denotes SE and “***” denotes *p* < 0.001.

Figure 2A shows the trial variation of odd sensation scores (mean ± SE across participants) of Group 1 (40 trials in the Escal-view) and Group 2 (2 trials in the Escal-view and then 38 trials in NoStripes-view). The odd sensation gradually decreased with trials, and disappeared in the late phase of trials in both groups. Figure 2B compares the odd sensation scores of the mean of the 23rd–27th trials (labeled as “Initial”) and the mean of the 56–60th trials (labeled as “Late”) of the two groups (G1 and G2). The two-way ANOVA indicated that the odd sensation scores significantly decreased with trials (*F*_(1,46)_ = 231, *p* < 2e-16), but no significant difference was found in the group factor (*F*_(1,46)_ = 2.37, *p* = 0.13) or in the interaction (*F*_(1,46)_ = 1.64, *p* = 0.21).

**Figure 3:**
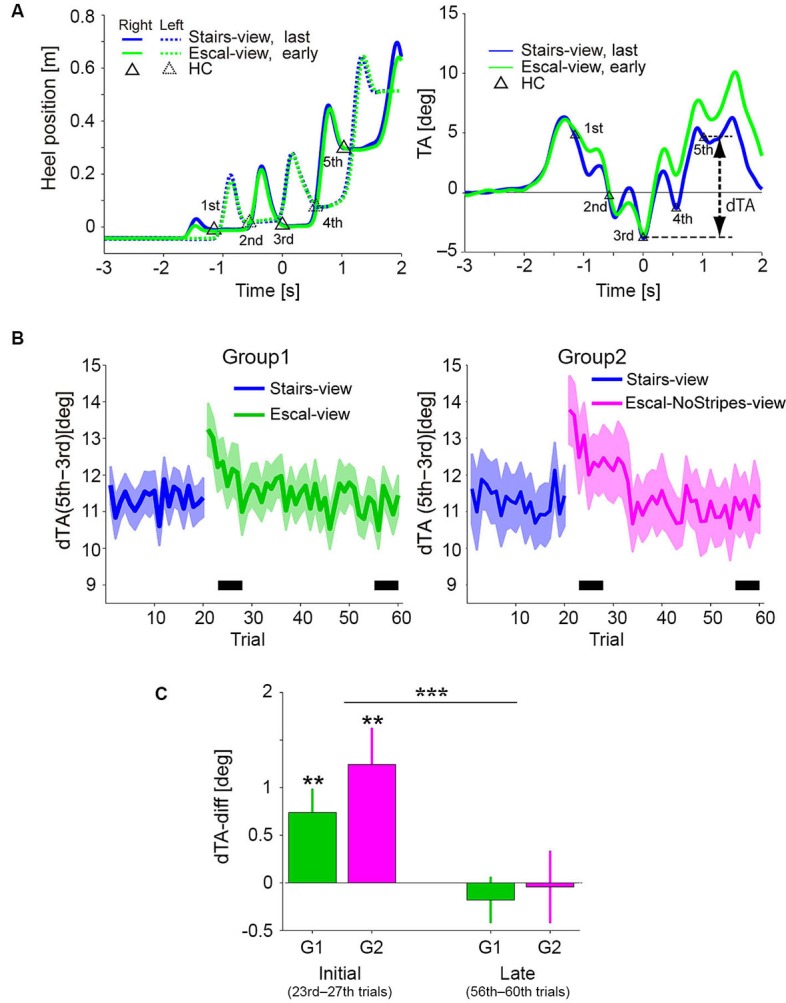
**(A)** Mean temporal patterns of heel positions (left panel) and TA (right panel) of the last epoch (16–20 trials) in the Stairs-view condition and of the following early epoch (21–25 trials) in the Escal-view condition for a particular participant. Triangles denote heel contacts (HCs) in the Stairs-view condition. The heel patterns look similar between the Stairs-view and Escal-view conditions, but the TA appeared to be different in these two conditions. **(B)** Trial histories of the dTA (difference between TAs of third and fifth steps) of the participants who felt an odd sensation (Solid line: mean. Colored area: SE. Left panel for Group 1 and right panel for Group 2). **(C)** Comparison between mean dTA-diffs (deviation of dTA from that in the Stairs-view condition) during the initial phase (23rd–27th) and late phase (56–60th), indicated by the thick horizontal bar in panel **B**, in both groups. Asterisks above the bar denote statistical significance of the dTA-diff from zero by *t*-test, and asterisks above the horizontal line denote statistical significance of the trial-phase (initial/late) factor, obtained by ANOVA. The error bar denotes SE and “**” *p* < 0.01, “***” *p* < 0.001.

Figure 3A shows the temporal profiles of the heel positions and TA (mean of 5 trials in each condition) in the Stairs-view (blue) and Escal-view (green) conditions of a particular participant of Group 1. Triangles in each graph indicate the corresponding data in the Stairs-view condition at the detected HCs of the first to fifth steps. While the temporal profiles of heel positions were almost identical in the Stairs-view and Escal-view conditions (left panel), the TA appeared to increase more in the Escal-view condition than in the Stairs-view condition after the third HC (right panel). This condition-dependent change of TA was commonly observed in Group 1 and Group 2 (left and right panels, respectively, in Figure 3B depicting the delta TA [dTA: TA(5th)–TA(3rd)] over the 60 trials in Experiment 1). As shown in both panels, dTA suddenly increased after the appearance of the escalator view (21st trial). Similar to the odd sensation shown in Figure 2A, dTA gradually decreased with trials (around trials 20–40) and almost returned to the value for the Stairs-view condition (blue curve) of each group.

Figure 3C shows the dTA-diff averaged around the initial (23rd–27th trial) and late (56–60th trial) phases in the Escal-view condition of Group 1 and the NoStripes-view condition of Group 2. Here, dTA-diff represents a deviation of dTA from that in the Stairs-view condition [i.e., (mean dTA of initial or late phase in the Escal-view or NoStripes-view condition) – (mean dTA of 11–20th trials in the Stairs-view condition)]. Note that, the first two trials in the Escal-view condition in both groups were excluded from the averaging for the initial phase analysis (see also Section Experimental Procedure). The dTA-diff of both groups were significantly positive in the initial phase (*t*-tests, *p* < 0.01 for both groups), but almost completely vanished in the late phase (*t*-tests, *p* > 0.4 for both groups). The two-way ANOVA indicated that the trial-phase factor (initial/late) was significant (*F*_(1,46)_ = 17.7, *p* < 0.001) but the group factor (*F*_(1,46)_ = 0.86, *p* = 0.36) and the interaction (*F*_(1,46)_ = 0.5, *p* = 0.48) were insignificant. The positive dTA-diff of Group 2 in the initial phase indicates that the postural forward sway was not eliminated by veiling the tread-board stripes.

We also analyzed the postural changes of the remaining participants (Group 3) who reported no odd sensation in the Escal-view condition. Mean dTA-diff of 23rd–27th trials was −0.089 (± 0.34 SE) deg and statistically insignificant (*t*-test, *p* = 0.8). This observation is consistent with the idea that the odd sensation is tightly coupled with the postural change that frequently occurs on a stopped escalator (Fukui et al., 2009).

### Effect of visual motion on postural change and odd sensation

After sufficient decays of the TA-change and odd sensation (Experiment 1), participants were asked to step onto the escalator mock-up with the VM of white disk patterns shown on the frontal and bilateral-side screens (see Section Experimental Procedure for details). We applied four VM conditions (NoVM, Cont, Expa, and Rand) as the participants stepped onto the escalator tread-boards.

**Figure 4:**
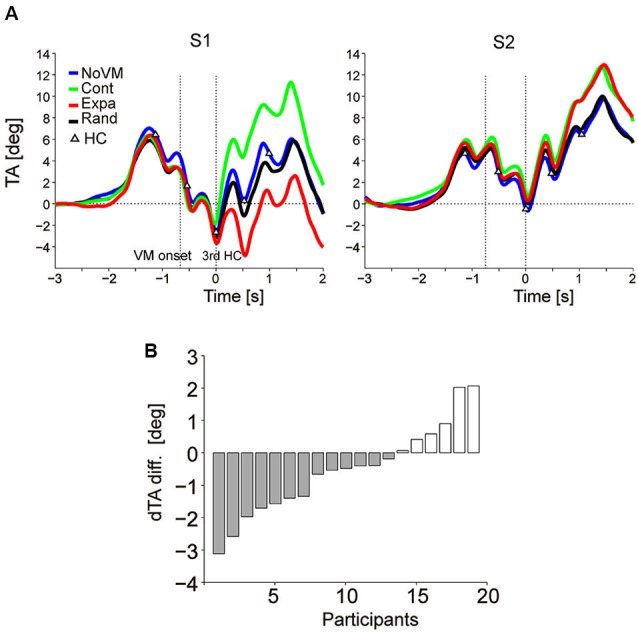
**Change in upper body TA caused by dynamic visual stimuli.** **(A)** Mean temporal patterns (first 10 trials for each stimulus condition) of TA of two participants (S1 and S2) in the four visual stimulus conditions. Visual motion (VM) onset and 3rd HC are indicated by vertical dotted lines. While additional TA increases were observed for the Cont stimulus in both participants (green curve), the TA decreased in S1 but increased in S2 for the Expa stimulus (red curve), relative to the TAs of the corresponding participants for the NoVM stimulus. A clear difference was not observed between the TA patterns for the NoVM (blue curve) and Rand (black curve) stimuli in the sampled participants. **(B)** Inter-subject variation of dTA-diffs for the Expa stimulus in Experiment 2, ordered by amplitude of dTA-diff.

Figure 4A shows the TA temporal patterns of two participants (S1 and S2) who showed an additional TA increase in the Cont condition (green curve) after time 0 (third step HC) compared to the TA pattern in the NoVM condition (blue curve) while those TA changes in the Expa condition (red curve) was completely opposite with each other (TA decreased in S1, but increased in S2, compared to that for the NoVM condition). Actually, for all 19 participants, dTA-diff [= dTA(Cont) – dTA(NoVM)] increased significantly (*t*-test, *p* < 0.0001) in the Cont condition, but consistent increase or decrease in dTA-diff in the Expa condition [= dTA(Expa) – dTA(NoVM)] was not seen across the participants (*t*-test, *p* = 0.11).

Figure 4B summarizes the dTA-diff values of all 19 participants in the Expa condition. Negative dTA-diff, as typically shown in S1 of Figure 4A, was observed in 13 participants (N-group) and positive dTA-diff, as typically shown in S2 of Figure 4A, was observed in six participants (P-group). For the N-group and P-group, we then separately analyzed the effects of different visual stimuli on the odd sensation score and the similarity to the odd sensation felt in the initial phase of the Escal-view condition.

**Figure 5:**
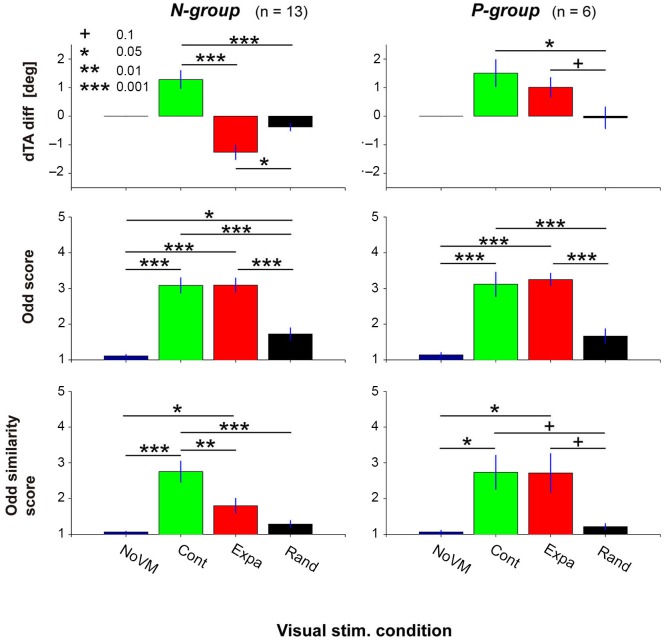
**Postural change (top panels), odd sensation (middle panels), and odd sensation similarity (bottom panels) of the N-group and P-group in the four visual stimulus conditions (NoVM, Cont, Expa, and Rand).** The N-group comprises participants who showed negative dTA-diff for the Expa stimulus; the P-group those who showed positive dTA-diff for the Expa stimulus. Each bar denotes the mean of the values of the first 10 trials in the corresponding condition, and its error bar denotes SE across participants. “+” *p* < 0.1, “*” *p* < 0.05, “**” *p* < 0.01, “***” *p* < 0.001.

Figure 5 summarizes the dTA-diff (top panels), odd sensation score (middle panels), and odd similarity score (bottom panels) in the four visual conditions for the N-group (left panels) and P-group (right panels). Each bar denotes the mean value across participants in each group. ANOVA results indicate that the visual stimulus condition significantly modulated dTA-diff (*F*_(2,12)_ = 31.9, *p* < 2e-7 for N-group; *F*_(2,5)_ = 6.02, *p* < 0.02 for P-group). While the postural change (dTA-diff) for the Expa stimulus (red bar) was different between the two groups, the postural change significantly increased for the Cont stimulus (green bar) in both groups, suggesting a consistent postural response across all participants. For the Rand stimulus, a small but significant postural change was observed in the N-group (*t*-test, *p* < 0.02) but not in the P-group (*p* = 0.88).

The visual stimulus condition also has an impact on the subjective odd sensation (*F*_(3,12)_ = 56.8, *p* < 1e-13 for N-group; *F*_(3,5)_ = 37.3, *p* < 4e-7 for P-group). As shown in the middle panels of Figure 5, a *post-hoc* comparison indicated that significantly higher scores were obtained both in the Cont and Expa conditions than in the NoVM condition in both groups. On the other hand, the odd sensation score in the Rand condition slightly increased in the N-group, but did not increase significantly in the P-group (*p* = 0.18), compared to the score in the NoVM condition. These results suggest that the odd sensation score tends to increase when postural change is greatly induced regardless of the VM direction.

As for the odd-sensation similarity score, the visual stimulus condition was also a significant factor (*F*_(3,12)_ = 18.9, *p* < 2e-7 for N-group; *F*_(3,5)_ = 5.48, *p* < 0.01 for P-group). A *post-hoc* comparison indicated that odd-sensation similarity score for the Cont stimulus was significantly higher than that for the Expa stimulus in the N-group, as shown in the bottom left panel of Figure 5, while this difference in the similarity scores was not significant (*p* = 0.99) in the P-group as shown in the bottom right panel. Additionally, the similarity scores of all participants in the three dynamic visual stimulus conditions (*n* = 56) significantly correlated with the dTA-diffs (*r* = 0.36, *p* < 0.005), while the odd scores did not (*p* > 0.3). The main reason for this insignificant correlation between the odd scores and dTA-diffs is that the high odd scores were obtained independently of the direction of dTA-diff (left top and middle panels in Figure 5). These results indicate that unexpected forward body sway itself is a critical factor in inducing an odd sensation similar to that felt for the escalator mock-up. Since the postural change characterized here was purely induced by exogenous VM stimuli, our data suggest that the endogenous escalator-specific motor program is not indispensable for inducing the odd sensation frequently felt on a stopped escalator.

## Discussion

The results clearly showed that for a majority of participants (86%), the postural change and odd sensation suddenly started in the initial trials when they stepped onto the escalator mock-up with the tread-board stripes and hand-rails unveiled. Since this was comparable to the 89% of our open-lab visitors (173/194) who reported the odd sensation experience upon stepping onto a stopped escalator, it is unlikely that the sampled population in this study was accidentally biased. These influences did not suddenly disappear even when the tread-board stripes were again occluded. Additionally, even after almost complete decay of the postural change and odd sensation with successive trials in the escalator mock-up condition, the postural change and odd sensation were once again successfully induced by VM. Here we will discuss the mechanisms generating the postural change and odd sensation.

### Postural change for the stopped escalator is not ascribed to the depth misperception

It has been shown that misperception of spatial attributes of external objects affects several motor tasks (Glover and Dixon, 2001; Smeets et al., 2002; Mendoza et al., 2006), although some classes of motor tasks are less influenced by perceptual illusions than others (Aglioti et al., 1995; Flanagan and Beltzner, 2000). For instance, arrow direction at a line tail induces biases in pointing location and grip aperture (Gentilucci et al., 1996; Westwood et al., 2000; Franz, 2001; Glazebrook et al., 2005), and the size of surrounding circles affects grip aperture for the center circle (Pavani et al., 1999; Franz et al., 2000). Likewise, as mentioned in the introduction, the depth misperception caused by the binocular miss-correspondence in the pattern of the tread-board stripes (Cohn and Lasley, 1990; Munck-Fairwood, 1992) could result in postural imbalance. If this were the case, one could expect sudden eliminations of the TA change and high odd sensation scores even with the tread-board surface patterns veiled. Our results, however, clearly negate this possibility since the postural change and accompanying odd sensation were not eliminated even after the periodical surface grooves of the tread-boards had been veiled.

One could imagine that the postural changes in the NoStripes-view condition were caused by an aftereffect of the first two trials of the Escal-view condition in Group 2, rather than by the effect of the escalator-specific habitual motor program. This is unlikely, however, because previous studies showed that the aftereffect of a similar postural control task quickly vanished after 2–3 trials (Bunday et al., 2006), and that the postural change and odd sensation were suddenly eliminated when the stair condition was inflicted just after the trials with a full escalator view (Fukui et al., 2009). Therefore, in addition to our previous examination of the escalator’s structural (inconsistent step heights) effect (Fukui et al., 2009), the present study strongly supports the idea that the endogenous escalator-specific motor program implicitly induces a postural change leading to the odd sensation.

### Slow adaptation of postural control to the escalator mock-up

A clear postural response was observed in many participants in the initial trial in the apparent escalator mock-up condition (Escal-view). This observation was completely different from many previous experiments (Anstis, 1995; Pelah and Barlow, 1996; Reynolds and Bronstein, 2003, 2004, 2006; Bunday et al., 2006; Bronstein et al., 2009) in which pre-adaptation trials/duration were given immediately before the test. This means that this escalator-view-dependent motor control is well learned in the real world and emerges with an escalator’s appearance. Here we should mention that, as shown in Figures 2, 3, it took over 10 trials to eliminate the postural change and odd sensation. This (de-)adaptation appears to require a much longer experience than the short-term adaptation (2–3 trials to adapt or de-adapt) demonstrated in riding a moving sled (Bunday et al., 2006), while behavioral changes against the participants’ conscious awareness were observed in both the moving sled and escalator mock-up (i.e., participants were clearly aware of the upcoming condition). Therefore, it seems to be hard to overcome the subconscious association between an escalator’s appearance and the motor program for escalator riding.

One could speculate that this relatively slow adaptation is simply due to the strength of the association between the sensory (visual) cue and motor program. In the context of motor learning studies, however, in addition to the remarkable features of reaction time (Saijo and Gomi, 2010) and inter-limb transfer (Malfait and Ostry, 2004), the differences in the amplitude of the aftereffect and speed of de-adaptation (Kagerer et al., 1997; Michel et al., 2007; Saijo and Gomi, 2010) indicate distinct mechanisms of learning and/or memory for motor control. Furthermore, several physiological studies (Kassardjian et al., 2005; Shutoh et al., 2006) have demonstrated that distinct neural mechanisms are involved in short- and long-term adaptations even in a particular single motor adaptation. Taken together, it can be inferred that the context-dependent postural change automatically triggered by the escalator’s appearance in Experiment 1 is configured through a long-term (habitual) adaptation that may be stored in a motor memory distinctive from the one for the short-term adaptation.

### Postural changes by visual motion when stepping onto an escalator mock-up

After the almost complete decay of the postural change and odd sensation with successive trials, we applied several patterns of sudden VM to induce postural changes when participants stepped onto the escalator mock-up. The reflexive compensatory postural responses induced by translational or contraction/expansion VM are well known (Lee and Lishman, 1975; Lestienne et al., 1977; Nashner and Berthoz, 1978; Berthoz et al., 1979; Masson et al., 1995). As in these studies, here we observed a significant change in forward postural sway across participants for the contracting VM (Cont stimulus). For the expansion (Expa stimulus), on the other hand, backward postural sway was not consistently observed across participants (dTA-diff < 0 for 13/19 participants, dTA-diff > 0 for 6/19 participants), unlike in most previous studies (Lestienne et al., 1977; Nashner and Berthoz, 1978; Berthoz et al., 1979; Masson et al., 1995). In the context of the influence of VM (optic flow) on standing postural readjustment, one exception (Baumberger et al., 2004) reported a forward postural shift measured by the center of pressure against the direction of an “approaching optic flow”, which corresponds to the expansion stimuli in the current study.

Here, it should be noted that the speeding up of expanding VM can be interpreted in two possible ways in the context of walking. One is that the flow speeds up as a result of a forward postural sway. The other is that the flow speeds up because of an increase in heading (forward walking) speed. If the former, it would be reasonable to generate a backward postural adjustment for stabilizing a posture. If the latter, however, generating a forward postural change would be reasonable in order to increase the postural stability against the increase of the speed of the forward transfer, as observed in previous studies (Reynolds and Bronstein, 2003; Fukui et al., 2009). Since the postural control is greatly dependent on the context of motor environments (Nashner, 1976; Bronstein and Buckwell, 1997), the participant-dependent postural sways for the expanding VM obtained in this study can be partly ascribed to the variation of the implicit interpretation of the stimulus across participants.

On the other hand, it might be difficult to interpret contracting VM as a speed reduction of a forward movement, since decreasing the speed of the movement does not produce the contracting VM. It would be rather reasonable to assume that contracting VM is produced by a backward body sway caused by, for example, slipping on a wet floor. It is therefore adequate that the forward sway was produced significantly across all participants as a compensatory postural response for avoiding the risk of falling down backward. Thus there would be less ambiguity of the interpretation in the contraction condition than in the expansion condition.

### Postural changes caused by implicit controls induced odd sensation

Regardless of the postural sway direction caused by the visual stimuli, the odd-sensation score was high when large sway was induced, as shown in Figure 5 (odd score). In addition, when a forward sway was induced by visual stimuli (Cont condition in the N-group and Cont and Expa conditions in the P-group shown in the top panels of Figure 5), the participants reported an odd sensation relatively similar to the one initially felt for the escalator mock-up.

One could think that the similarity score was not extremely high even in the Cont condition. We speculate that a possible reason of this modest score is an insufficient similarity of the behavioral changes in the VM conditions to those in the stopped escalator condition. Specifically, our VM stimulus does not induce the leg-behavioral changes frequently observed in the stopped escalator condition (Fukui et al., 2009). Actually, in the free-form report obtained after the experiment, half of the participants (9/19) reported some strange leg behavior in the initial escalator mockup condition, but none of them reported it in the VM conditions. Another possible reason is an insufficient excitation of postural change (postural change was about 1 deg for Cont in Figure 5, but over 2 deg for the very first trial in the Escal-view condition in Figure 3B). This smaller postural change in the VM condition could degrade the score of odd sensation similarity. In spite of these insufficiencies, the significant correlation between similarity scores and body tilts (see Section Results) and tight linkage between odd sensation and the forward body tilt, found previously (Fukui et al., 2009) would strongly suggest that the odd sensation induced by the contraction VM is similar to that induced by the stopped escalator.

In the free-form reports about the peculiar bodily sensation after all experiments, many participants in Experiment 2 reported a sensation of unintended forward tilt for the Cont stimulus condition (18/19) as well as for the Escal-view condition in Experiment 1 (14/19), while sensation of unexpected backward tilt (a strange pushing/pulling sensation in the backward direction) was frequently reported as an odd bodily sensation for the Expa stimulus condition (16/19). For the random motion stimulus, none of the participants reported a forward- or backward-body-tilt sensation. These reports would also suggest that the differences in the odd sensation similarity scores for the different visual stimulus conditions were associated with postural changes.

### Odd sensation may stem from a lack of self-motor prediction

Why was the odd sensation induced by the VM (Experiment 2) similar to that induced by the escalator mock-up (Experiment 1)? To answer this question, we need to recall that the sources inducing the postural forward sway were completely different in Experiments 1 and 2: one was an endogenous trigger (implicit perception that evokes escalator-specific habitual motor program) and the other was an exogenous trigger (visual stimulus that produces reflexive postural response). As described in the introduction, the former could induce the high-level conflict between the conscious awareness and implicit perception for motor control, but the latter could not. On the other hand, a common aspect of the phenomena caused by these two triggers was an inappropriate postural change produced by self-produced motor commands without conscious motor intention. This suggests that such an unintentional and subconsciously induced postural change itself, rather than the nature of the above triggers, appears to be essential in characterizing the similar odd sensations.

The next question is, why do the unintentional behavioral changes induce the odd sensation? To examine this question, we may need to understand how we discriminate our own motor action from externally generated action. Previous studies reported sophisticated experimental results indicating that sensory information caused by self-generated movement is canceled out by using a copy of a motor command, called an efference copy (von Holst, 1954) or collorary discharge (Sperry, 1950). This idea has been extended to a precise computational model, a forward prediction hypothesis (Wolpert et al., 1998; Wolpert and Ghahramani, 2000), in which the upcoming movement states are predicted by copies of motor commands. Sensory suppression by self-generated movements (Blakemore et al., 1998) and enhancement of self-recognition (Tsakiris et al., 2005) have been nicely explained by the forward prediction model.

According to this theory, if a copy of the final motor command had been utilized in predicting the movement outcomes, the postural sways (i.e., motor errors) triggered by the escalator mock-up view or by the surrounding VM could be properly attributed to self-generated action. However, unlike inaccurate voluntary movements that sometimes occur in daily life, these postural sways appeared not to be perceived as a self-generated action. We speculate, therefore, that the self-motor prediction, which might be processed by front-parietal network (Sirigu et al., 2004; Farrer et al., 2008), is inappropriate for the implicitly induced body tilts shown in this study. For a well-learned voluntary movement, it has frequently been postulated that motor prediction by an internal model is driven by an action intention (Jeannerod, 1995; Wolpert and Ghahramani, 2000). In contrast to such intended action, involuntary responses externally caused by a stimulation of the corticofugal pathway seem to be less attributable to the self-action (Gandevia, 1987; Haggard and Clark, 2003; Voss et al., 2006).

It can therefore be inferred that motor commands without conscious intention contribute less to the generation of motor predictions since the resultant responses usually act to implicitly assist voluntary actions or to automatically regulate posture if they work appropriately. This lack or insufficiency of the prediction for implicit motor control would lead to a failure in attributing inappropriate behavioral changes to self-generated action. On the other hand, our consciousness could not ascribe the postural imbalances to the external events, such as the escalator’s appearance and VM, because unnatural postural changes induced by these stimuli are rarely experienced. As a result, behavioral error (postural change) could not be assigned to an appropriate cause. In other words, a conflict between the intended action and sensory events of postural change resulted by the implicit motor control would be occurred in this situation. Since error assignment is crucial in any kind of motor regulation and learning in human life, the odd sensation seems to be a fundamental alert for conscious awareness to inform us of a violation of or conflict in motor error assignment. The neural basis of this peculiar sensation has not yet been understood, but the sensorimotor error detection and conflict monitoring networks (i.e., anterior cingulate and dorsolateral prefrontal cortex) (Carter et al., 1998; Botvinick et al., 1999, 2004; Fink et al., 1999; Gehring and Knight, 2000; Garavan et al., 2003) could contribute in yielding the sensation because of some similarities in the conflicting relationship between conscious intension and sensorimotor error. Further examinations would be of particular interest in revealing the interplay among the implicit motor process, prediction, and conscious sensation.

## Conflict of interest statement

The authors declare that the research was conducted in the absence of any commercial or financial relationships that could be construed as a potential conflict of interest.
